# Fluorescent Investigation of Proteins Using DNA-Synthetic Ligand Conjugates

**DOI:** 10.1021/acs.bioconjchem.3c00203

**Published:** 2023-08-09

**Authors:** Lulu Winer, Leila Motiei, David Margulies

**Affiliations:** Department of Chemical and Structural Biology, Weizmann Institute of Science, Rehovot, 76100, Israel

## Abstract

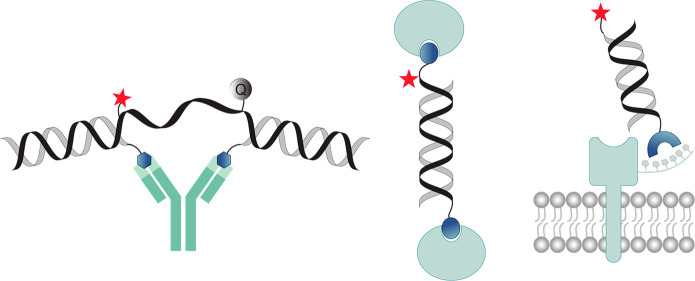

The unfathomable role that fluorescence detection plays in the life sciences has prompted the development of countless fluorescent labels, sensors, and analytical techniques that can be used to detect and image proteins or investigate their properties. Motivated by the demand for simple-to-produce, modular, and versatile fluorescent tools to study proteins, many research groups have harnessed the advantages of oligodeoxynucleotides (ODNs) for scaffolding such probes. Tight control over the valency and position of protein binders and fluorescent dyes decorating the polynucleotide chain and the ability to predict molecular architectures through self-assembly, inherent solubility, and stability are, in a nutshell, the important properties of DNA probes. This paper reviews the progress in developing DNA-based, fluorescent sensors or labels that navigate toward their protein targets through small-molecule (SM) or peptide ligands. By describing the design, operating principles, and applications of such systems, we aim to highlight the versatility and modularity of this approach and the ability to use ODN-SM or ODN-peptide conjugates for various applications such as protein modification, labeling, and imaging, as well as for biomarker detection, protein surface characterization, and the investigation of multivalency.

## 1 Introduction

The use of fluorescence as a means to investigate biomolecules has become a go-to practice in countless laboratories, considering its high sensitivity, minimal functional interference, and temporal stability.^1,2^ Owing to their critical role in health and disease, proteins are often the biological target at the heart of many fluorescence-based studies.^3^ By using fluorescence labeling or sensing methods one can gain substantial insights into the properties of a wide range of proteins of interest (POIs), such as their expression levels, cellular localization, and conformation, as well as their enzymatic activity and ability to engage in binding interactions.^4−11^ The immense versatility and usefulness of fluorescence-based labeling and detection methods have sparked interest in simplifying the task of labeling proteins and expanding the scope of detection to more complex environments and protein-mediated biological processes.^12−14^

A common approach for the fluorescent labeling of proteins is fusing them to fluorescent protein tags.^15−17^ However, this method is often hindered by the need to genetically engineer the POI, as well as by the relatively large size of fluorescent proteins, which may impair POI function. An alternative, extensively used way to label proteins utilizes synthetic probes that integrate a specific protein binder and a small-molecule (SM) fluorophore,^12,18−24^ facilitating the labeling of both engineered and nonengineered POIs through the formation of either covalent or noncovalent probe–protein interactions. If such binding events result in specific fluorescence responses, e.g., the generation of a turn-on emission signal, then these chemical probes can be utilized as sensors.

When aiming to achieve a simple, modular, and versatile approach to investigating proteins with fluorescent chemical probes, it is highly important to consider the molecular scaffolds used to generate them. In addition to controlling the number of protein binders, dyes, and the distance between them, scaffolds affect various essential features of the probes, such as their water solubility, ease of synthesis, and ability to form well-defined nanostructures as well as produce a fluorescent response. Fulfilling the above-mentioned requirements, oligodeoxynucleotides (ODNs) have emerged as promising building blocks for scaffolding fluorescent probes for protein labeling, sensing, and imaging.^25,26^

In this review, we aim to provide a broad scope of versatile fluorescent platforms for protein investigation that share the common theme of combining SM- or peptide-based ligands together with DNA constructs. This integration significantly expands the chemical space available for investigating proteins using fluorescent DNA probes. Additionally, this approach facilitates precise targeting of well-defined active sites or small affinity tags, which have rarely been targeted by other DNA-based protein binders such as aptamers.^27^ In that respect, fluorescent systems that contain aptamers as recognition elements,^28^ albeit prominent, will not be covered in this review, as well as platforms that utilize outputs that differ from fluorescence.^26,29,30^

Two distinct approaches for targeting proteins with DNA-synthetic ligand conjugates are discussed in this review: Section 2 focuses on studies where the POI is targeted outside the ligand binding domain or catalytic site and thus retains its activity. Conversely, section 3 delves into the utilization of fluorescent probes that occupy the binding site, rendering it inactive but nonetheless facilitating unique applications. Ranging from site-specific labeling to the sensing of clinically relevant targets, this review will discuss important achievements and diverse viewpoints by describing various landmark studies.

## 2 Probes That Bind Outside the Ligand Binding Domain

**Figure 1:**
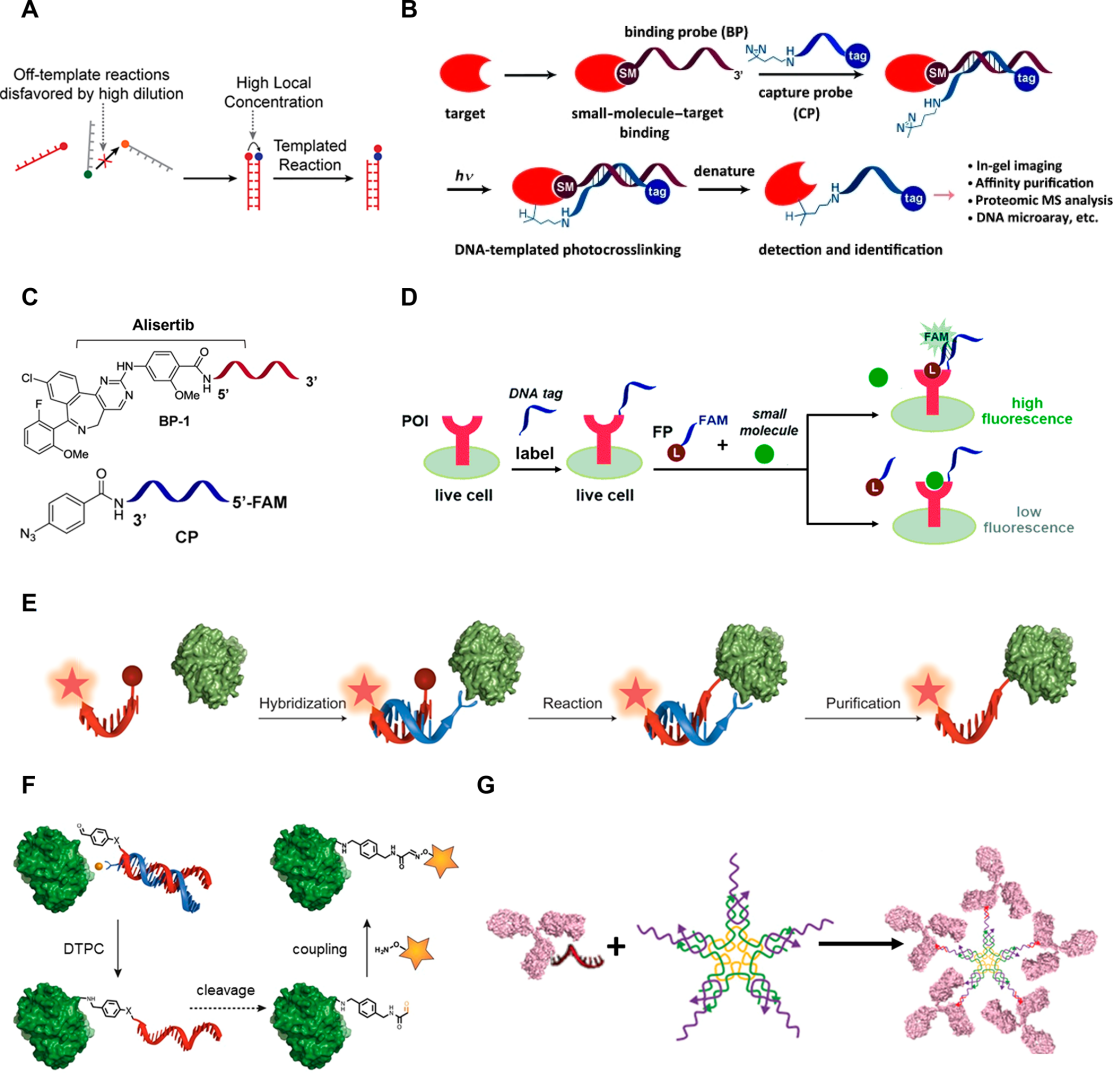
A. Basic principles of DTS: nucleic acid hybridization promotes the proximity between specific building blocks, followed by their subsequent reaction. Adapted with permission from ref (50), published by the American Chemical Society and licensed under Creative Commons 4.0 (CC 4.0) series. B. Operating principles of DPAL, a dual-probe system for labeling a ligand-binding protein. Reproduced with permission from ref (37). Copyright 2013 John Wiley & Sons, Inc. C. Strands used in DPAL-based identification of Alisertib protein targets. Adapted with permission from ref (39). Copyright 2017 John Wiley & Sons, Inc. D. Live-cell screening of membrane protein binders using DPAL. Adapted with permission from ref (42). Copyright 2021 The Royal Society of Chemistry. E. General concept of DTPC, an affinity-tag-guided method for covalent protein labeling. Adapted with permission from ref (47). Copyright 2014 Springer Nature. F. Obtaining a DNA-free fluorescently labeled protein. Adapted with permission from ref (48). Copyright 2016 John Wiley & Sons, Inc. G. The formation of IgM-like nanostructures using peptide-directed DTPC. Adapted with permission from ref (49). Copyright 2019 John Wiley & Sons, Inc.

One reason to append an ODN-synthetic ligand conjugate with a fluorophore is simply to determine whether a protein was successfully targeted (and often labeled) by the designated probe. Another common motivation for using such systems is the investigation of various protein properties such as structural features, expression levels, or localization as well as their engagement in binding interactions. Because many of these applications require the targeted protein to remain active, it is preferable to direct probe binding away from domains essential for function such as ligand binding domains (LBDs) or catalytic sites of enzymes. Such nondisruptive labeling has been achieved with SM-based fluorescent probes that can bind an artificially fused peptide (or a small protein)^31−33^ or with probes that can conjugate a fluorescent dye outside the LBD.^34^ Among the advantages of generating similar probes from DNA is the ease of synthesis and assembly, along with the ability to obtain nondisruptive labeling^35^ using the principles underlying DNA-templated synthesis (DTS, Figure 1A),^36^ in which nucleic acid hybridization promotes the proximity between specific building blocks, encouraging their subsequent reaction.

### 2.1 Nondisruptive Labeling of Native (Nonengineered) Proteins

In 2013, Li and co-workers introduced a DTS-based method for labeling SM-binding proteins. This method, termed DPAL (DNA-programmed photoaffinity labeling),^37^ employs two modified strands (Figure 1B): one is the binding probe (BP), modified with an SM ligand. The second is a complementary strand, appended with a tag (fluorophore or biotin) and a photo-cross-linker, forming the capture probe (CP). Covalent fluorescent labeling of a protein is achieved by incubating it with the BP, followed by CP hybridization, UV irradiation, and last, denaturation of the BP-CP duplex. The Li team suggested that the CP is, in principle, universal and can be combined with a library of diverse BPs, providing modularity and multiplexity. Additional optimization of DPAL produced a significant increase in efficiency, facilitating the labeling of low-abundance proteins in complex mixtures such as cell lysates.^38^

DPAL was further used by Wang et al. to discover novel protein targets of the drug candidate Alisertib by appending the CP with a phenyl azide cross-linker and a 5′-fluorescein (Figure 1C) followed by in-gel fluorescence.^39^ A similar application of DPAL was demonstrated by Bai et al., who developed a BP based on an ODN-peptide conjugate to label and profile proteins recruited by histone post-translational modifications.^40^ Recently, DPAL was combined with a DNA-encoded chemical library to screen for potential ligands of endogenous membrane proteins in living cells.^41^ Although this approach led to the identification of novel SM binders, one major limitation was the requirement to conjugate all candidate SMs to the strands. To address this, the Li team presented a fluorescence-based screening method to detect nonconjugated SMs. (Figure 1D).^42^ Briefly, a membrane POI is covalently labeled with a DNA tag using DPAL, and a complementary fluorescent strand (FP) is added. The FP is also appended with a known ligand, thereby occupying the LBD and fluorescently labeling the POI, whereas displacement of the FP by potential hits results in a decrease in fluorescence.

### 2.2 Nondisruptive Labeling of Proteins Fused to Affinity Tags

The systems described above (Section 2.1) were designed to target proteins with well-defined binding sites for known SM- or peptide-based ligands. Therefore, they are unsuitable for labeling, sensing, or imaging a wide range of proteins for which there are no available ligands. An alternative approach for labeling such proteins with fluorescent molecular probes is to fuse them to an unnatural affinity tag, to which the probes can be guided and selectively attach.^43−45^ Based on this concept and using different affinity tags, several ODN-based fluorescent probes have been developed.

#### 2.2.1 Nondisruptive Labeling of Isolated Proteins

The hexahistidine tag (His-tag) is one of the most prominent affinity tags used in recombinant protein expression and cell biology. An early demonstration of fluorescently labeling a His-tagged protein using modified ODNs was introduced by Allbritton and co-workers, who used a single fluorescent strand modified with both a His-tag-binding moiety, the nitrilotriacetic acid (NTA), as well as a photo-cross-linker.^46^ Although a covalently labeled active enzyme was afforded, one drawback was limited modularity, as a change in the fluorescent dye requires the synthesis of a new labeling strand. To address this problem, the Gothelf group introduced a method termed DNA-templated protein conjugation (DTPC), also utilizing the His-tag for covalent protein labeling (Figure 1E).^47^ In their multistrand approach, one strand is modified with an NTA while a complementary, fluorescent strand is appended with an activated *N*-hydroxysuccinimide (NHS) ester, prone to react with the side chain of proximal lysines. After establishing the principles of DTPC with a model His-tagged GFP, the Gothelf group also demonstrated the labeling of nonengineered metal binding proteins without any loss of activity. DTPC was further improved by replacing the unstable NHS ester with an aldehyde (Figure 1F), thereby reducing the probability of misguided labeling.^48^ Additionally, by installation of a cleavable linker, the attached strand can be cleaved off, thus leaving a traceless functional handle on the protein for further modification. The applicability of the improved DTPC method was demonstrated by generating an IgM-mimic antibody using Immunoglobulin G (IgG)-binding peptides that direct the labeling of IgG1 antibodies (Figure 1G), impressively retaining their antigen binding capacity in cell-based experiments.^49^ The Gothelf group indicated that this method provides a new platform for generating engineered IgMs, allowing up to five different antibodies to be assembled into a multiaffinity pseudo IgM.

#### 2.2.2 Nondisruptive Labeling of Cell Surface Proteins

**Figure 2:**
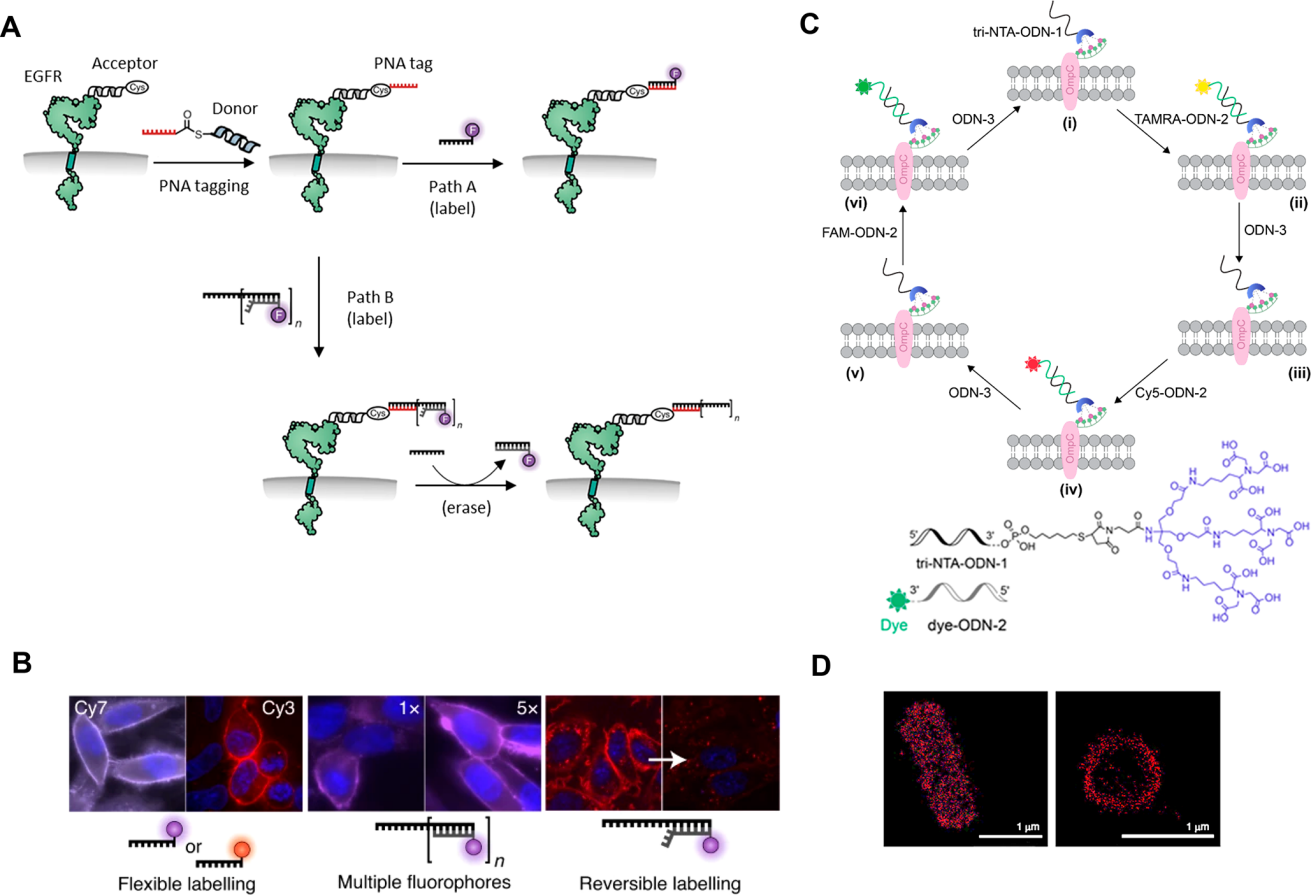
A. Live-cell covalent labeling of membrane POIs. B. Wide-field fluorescence microscopy images depicting adaptable (left), sensitive (middle), and erasable (right) labeling. Reproduced with permission from ref (51). Copyright 2021 Springer Nature. C. Dynamic labeling of the bacterial OmpC, initiated by the attachment of tri-NTA-ODN-1 to a His-tag. D. Super-resolution images of Cy5-decorated bacteria (C, state (iv) using stochastic optical reconstruction microscopy (STORM). Left: whole bacteria. Right: transverse cut. Reproduced and adapted with permission from ref (54), published by Springer Nature and licensed under Creative Commons 4.0 (CC 4.0) series.

Recently, the Seitz lab introduced a method for live-cell covalent labeling of cell surface proteins (CSPs), for instance, the epidermal growth factor receptor (EGFR) (Figure 2A).^51^ This approach is based on the affinity between pairs of coiled-coil-forming peptides (E3/K3 or P1/P2). First, the CSP of interest is genetically modified with both an acceptor peptide, e.g., E3, and an N-terminal cysteine (Cys-E3). Treatment of cells with a peptide nucleic acid (PNA) strand conjugated to the donor K3 peptide (PNA-K3) through a thioester bond leads to the attachment of the two coiled-coil-forming peptides. Covalent tagging of the CSP by the PNA strand is then facilitated by a proximity-induced native chemical ligation reaction. The PNA arm serves as a “landing platform”, readily available for subsequent fluorescent labeling by nucleic acid hybridization. The variability of this labeling method was demonstrated by labeling CSPs with multiple different dyes, as well as by displacing fluorescent strands from the CSP and relabeling it with different dye-appended strands (Figure 2B).

The main reason for covalently attaching fluorescent probes to a POI, as demonstrated in the systems previously described, is to prevent probe release due to subsequent washing or dilution steps. Alternatively, enhancement of binding affinity can also be achieved by multivalency.^52^ For example, it has been shown that introducing tri-NTA functionalities into His-tag labeling probes enhances their binding affinities and, consequently, their labeling efficiency.^53^ Using a tri-NTA-ODN conjugate, the Margulies team developed an efficient platform for noncovalent fluorescent labeling of His-tagged CSPs using a wide range of fluorescent dyes (Figure 2C).^54^ In this system, a tri-NTA-appended strand (tri-NTA-ODN-1) serves as an anchor for attaching a complementary fluorescent ODN (dye-ODN-2). Structurally, dye-ODN-2 is elongated with an overhanging region (or a “toehold”), which enables its detachment by a third complementary ssDNA (ODN-3). This system enabled the reversible labeling of the His-tagged outer membrane protein C (His-OmpC) of *E. coli* using various fluorescent dyes, as well as imaging the bacterium with super-resolution (Figure 2D).

#### 2.2.3 Glycoform Differentiation by a Pattern-Generating Protein Surface Sensor

**Figure 3:**
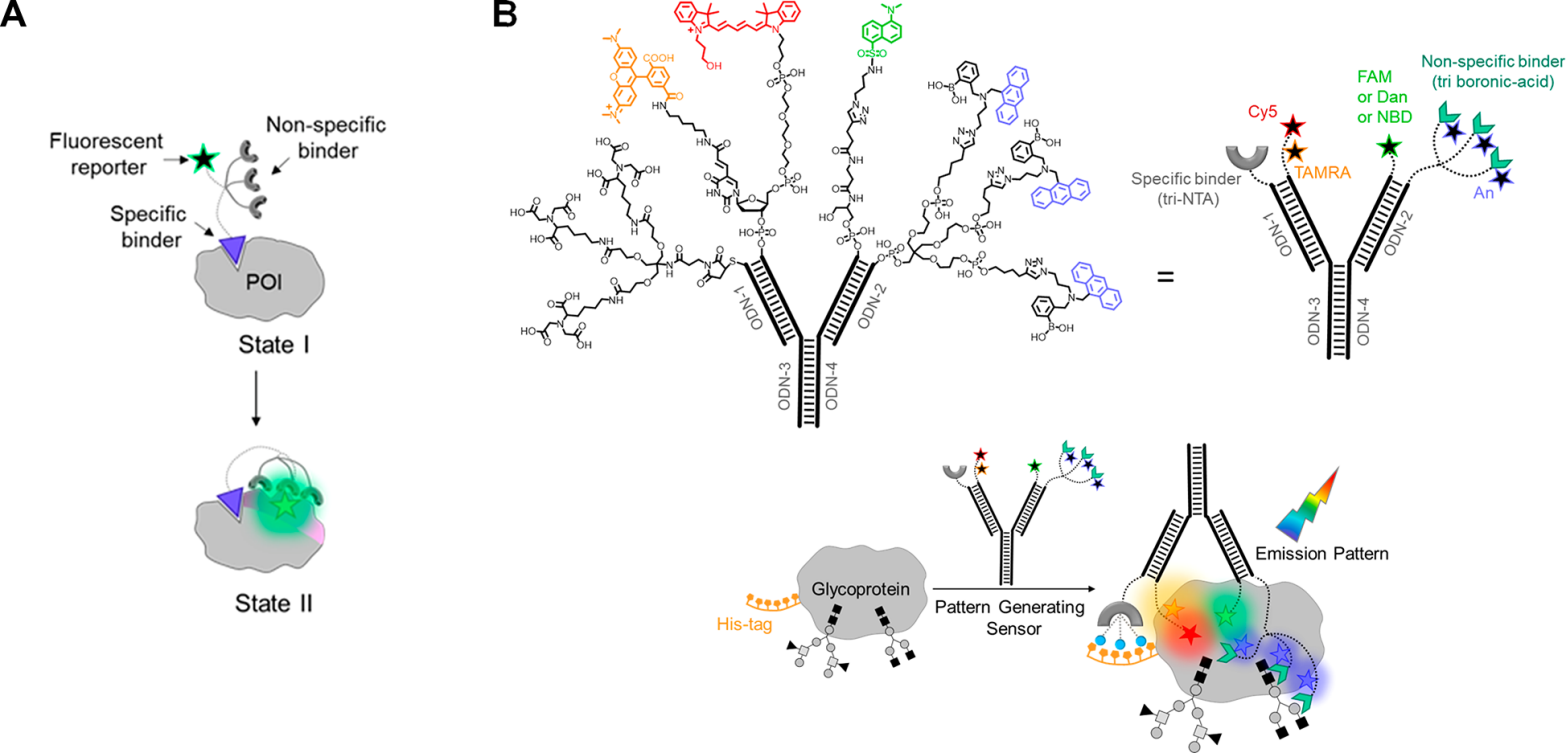
A. Schematic representation of a fluorescent sensor integrating a specific ligand of a POI along with nonspecific surface binders. B. Molecular design (top) and suggested mechanism (bottom) of a pattern-generating sensor able to identify protein glycosylation states. Adapted with permission from ref (58). Copyright 2020 American Chemical Society.

In the systems discussed so far (Figures 1 and 2), DNA-ligand conjugates were fluorescently modified in order to confirm the attachment (or conjugation) of the probe to the POIs, as well as to image them in their biological environment. However, some important structural properties of proteins cannot be determined with such simple labeling systems. For example, such systems cannot generally detect post-translational modifications located on solvent-exposed domains outside the LBD. To address this, the Margulies team has developed fluorescent molecular sensors that integrate both specific and nonspecific protein binders.^55,56^ The specific binders can interact with the POI (either genetically modified or native) with high affinity (Figure 3A, State I), while the nonspecific binders are synthetic agents that can engage in relatively weak, nonselective interactions with its surface (Figure 3A, State II). With such sensors, specific binding of the ligand to its protein target induces nonspecific interactions between the low-affinity binders and the POIs surface, resulting in a fluorescent response. The latter, proximity-induced interactions are somewhat similar to the ligand-directed interactions previously discussed (Section 2.1, e.g., Figure 1B).^57^ The key differences between these systems are that the protein surface sensors do not covalently label the POI, and the noncovalent interactions are accompanied by a change in the emission signal, enabling these sensors to detect subtle changes that occur on protein surfaces.

This approach was further used to develop pattern-generating DNA sensors that can straightforwardly discriminate between different glycosylation states of the therapeutically relevant glycoprotein target, human chorionic gonadotropin (Figure 3B).^58^ By using such sensors, which combine principles of protein surface recognition (Figure 3A) and pattern-based detection,^59,60^ distinct glycoform populations can be identified in a single fluorescence measurement. The design includes four strands (Figure 3B, top, ODNs 1–4) whose self-assembly enables facile integration of both specific and nonspecific protein binders, as well as distinct fluorescent reporters. ODN-1 bears a specific binder, tri-NTA, while ODN-2 is appended with a general glycan binder consisting of three copies of the well-known anthracene-boronic acid (An-BA) sensors^61,62^ that fluoresce upon saccharide binding.^63^ The dye-containing strands, ODN-3 and ODN-4, integrate into the system a wide range of fluorophores such as FRET (Förster resonance energy transfer) donors and acceptors and solvatochromic dyes. Self-assembly of the four strands into a Y-shaped scaffold endows the system with directionality and binding cooperativity, resembling bivalent antibody–antigen (Ab-Ag) interactions. The specific interaction with the His-tag promotes nonspecific binding to the glycosylated surface, consequently changing the local environment of the reporter dyes and resulting in unique fluorescent fingerprints for different glycoforms (Figure 3B, bottom). Such an approach obviates the need for glycan cleavage and chromatography, which are required in glycoprotein characterization by mass spectrometry.

## 3 Probes That Occupy the LBD or Antigen-Binding Site

A common feature of the platforms described in Section 2 is that POIs remain active following the initial interaction of the fluorescent probes with the LBD (Section 2.1) or affinity tags (Section 2.2). However, the retention of protein function is not essential for some applications, for example, the detection of protein biomarkers in biofluids or the *in vitro* characterization of protein–ligand or Ab–Ag interactions. Therefore, for such applications, ODN-ligand conjugates that remain bound to the LBD or Ag binding site can be used.

### 3.1 Turn-On Sensors for Detecting Proteins or Protein–Ligand Interactions

The ability to rapidly sense specific POIs, especially in complex biological mixtures, is highly sought-after in various research fields such as medical diagnosis and drug discovery.^64^ A key difference between sensing and labeling is that with sensors, the interaction with the POI is accompanied by a change in the emission signal, preferably a “turn-on” response. This abrogates the need to remove unbound probes by excessive washing steps, affording straightforward quantification. In many fluorescent ODN sensors, protein recognition motifs are based on DNA or RNA structures such as aptamers or sequences recognized by DNA-binding proteins.^65−67^ Other studies, however, demonstrate the power of using ODNs conjugated to SM or peptide-based ligands to afford turn-on protein detection.

**Figure 4:**
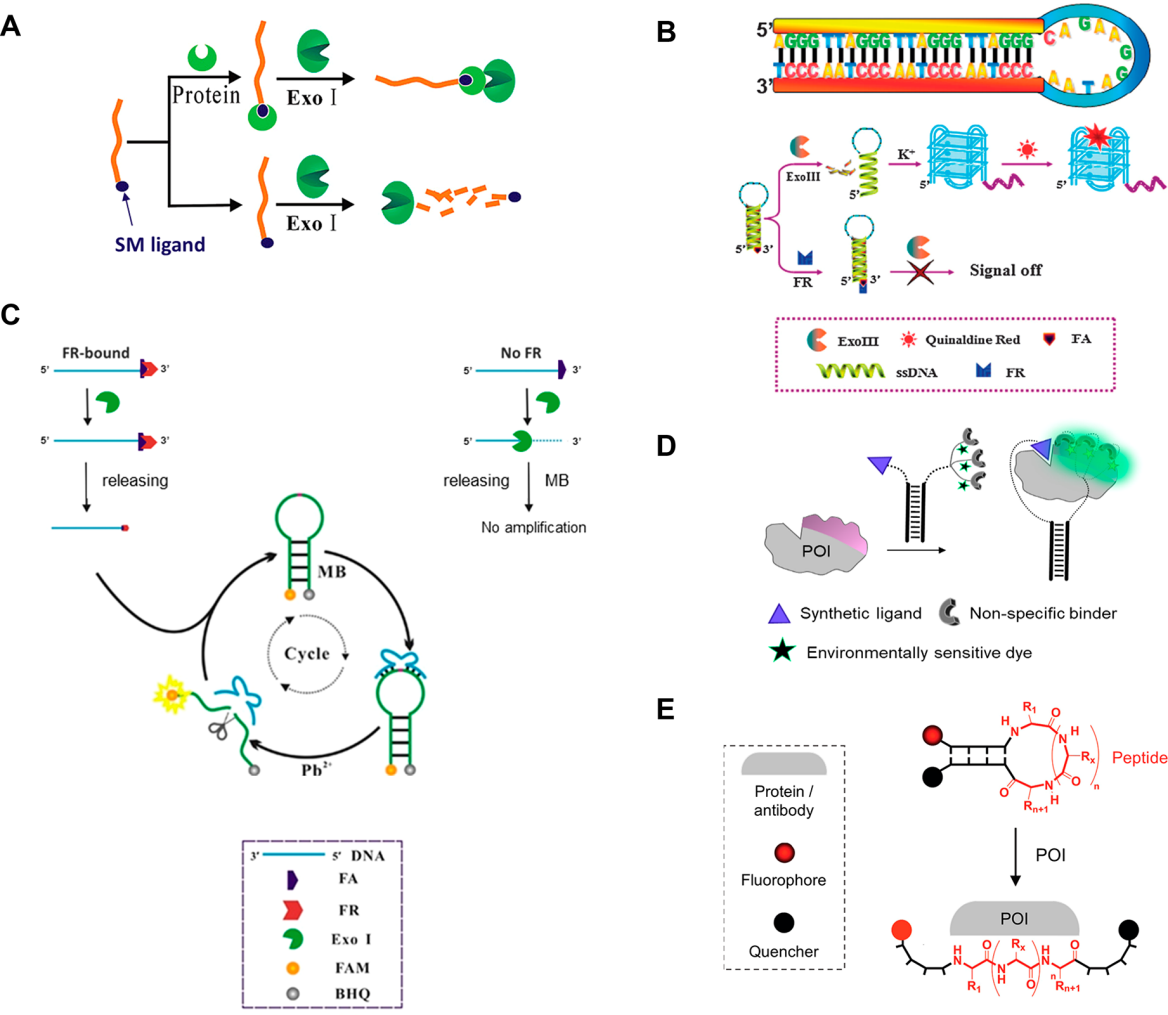
A. General principles of the terminal protection assay, in which SM-linked ssDNA is protected from hydrolysis while bound to a protein target. Adapted with permission from ref (68). Copyright 2009 American Chemical Society. B. Structure (top) and operating principles (bottom) of a fluorescent sensor for FR. Adapted with permission from ref (69). Copyright 2012 Royal Society of Chemistry. C. Schematic illustration of an FR biosensor with fluorescence amplification capabilities. Adapted with permission from ref (73). Copyright 2017 Elsevier. D. Operating principles of a turn-on fluorescent probe, exhibiting enhanced emission upon direct contact of an environmentally sensitive dye with the POI surface. E. Detection of protein targets by hairpin-like structures, stabilized by the hybridization of short PNA segments. Adapted with permission from ref (78). Copyright 2007 American Chemical Society.

One strategy for sensing proteins using fluorescent ODN-SM conjugates relies on the ability of exonucleases (Exos) to cleave DNA from the termini of oligonucleotide chains (Figure 4A).^68^ In the absence of a protein target, the ssDNA chain of an ODN-SM conjugate is degraded by Exos. However, the attachment of a protein to its ligand prevents the binding of Exos and the consequent DNA cleavage. This concept was initially implemented using an electrochemical readout to discriminate between the intact and degraded states^68^ and was later extended to rely on fluorescence. In one example, the folate receptor (FR) was detected by a system consisting of a quadruplex-responsive quinaldine red dye and a guanine (G)-rich hairpin structure appended with folic acid (FA) (Figure 4B).^69^ In the absence of FR, the hairpin is hydrolyzed by ExoIII from its 3′, halting at the loop region since its catalytic activity is specific to dsDNA. This digestion event induces the formation of a G-quadruplex to which the dye binds, resulting in a turn-on signal. In contrast, in the presence of FR, the cleavage of the hairpin is suspended, and the emission remains low. In two related systems for the detection of streptavidin (SA), ssDNA degradation was promoted by either ExoI alone^70^ or by combining both ExoI and ExoIII.^71^ By using similar principles and by incorporating quantum dot-ruthenium complexes into the design, a dual-color sensor for SA detection was also developed.^72^

To enhance the fluorescent response, a method that utilizes catalytic amplification was developed (Figure 4C).^73^ Signal amplification was achieved by using a DNAzyme conjugated to FA, together with a molecular beacon (MB) as the cleavable fluorogenic substrate. Binding of the FA-modified DNAzyme to FR protects it from being degraded by Exos, retaining its catalytic activity. Similar amplifiable systems were recently developed, distinguished by their “label-free” setup.^74,75^ Instead of using fluorophore-DNA conjugates, these platforms implement G-triplex-based MBs to generate fluorescent output. In a different catalytic amplification sensory system, protein detection was mediated by the CRISPR-Cas system.^76^ In this system, a DNA-SM conjugate is free to hybridize with a complementary crRNA in the absence of the POI, thereby activating the Cas12a enzyme and leading to the cleavage of a fluorophore–quencher pair. This work demonstrates that probes based on DNA-SM conjugates can also work in tandem with other unrelated biological machineries.

Fluorescence enhancement resulting from direct contact with a dye was also demonstrated (Figure 4D).^58,77^ Such sensors, in principle, are based on duplexes generated from two modified ODNs. One strand is appended with a synthetic ligand, whereas the second bears one or several environmentally sensitive dyes. With such systems, the binding of a synthetic ligand to the POI results in an induced proximity between proteins and dyes, changing the molecular environment of the dyes and leading to a turn-on fluorescence response. The way that this approach has been utilized to discriminate between protein isoforms or glycoforms via pattern recognition is discussed in Sections 3.3 and 2.2.3, respectively.

Lastly, protein detection was also achieved by inserting a peptide ligand into a PNA-based MB.^78,79^ At the center of such probes, a POI-binding peptide sequence is flanked by two complementary PNA arms, allowing for the formation of a stable hairpin structure (Figure 4E, top). In the absence of the POI, the proximity between the fluorophore and the quencher results in low emission. However, binding of the POI to the peptide leads to the opening of the structure and an enhanced fluorescence signal (Figure 4E, bottom). The Seitz group utilized such probes, which they termed hairpin peptide beacons (HPBs), to detect the SH2 domain of the Src-kinase.^78^ In this particular system, quenching relies on the alignment of two terminal pyrenes in the hairpin structure, leading to a preferred excimer emission. POI binding results in the separation of the two pyrenes and increased monomer emission. The principles underlying these systems can also be applied to antibody detection,^79^ as will be described in the next section.

### 3.2 Sensing of Antibodies and Antibody–Antigen Interactions

Antibodies (Abs) constitute a large class of clinically relevant proteins whose rapid and qualitative detection has the potential to streamline diagnostic efforts and improve patient care. However, methods for detecting specific antibodies mostly rely on laborious multistep processes such as ELISA or immunoprecipitation. Driven by the need for a simpler yet sensitive detection platform, researchers have explored the potential of using fluorescent ODN-Ag conjugates for the straightforward sensing of specific Abs.

An elegant demonstration of detecting anti-HIV Abs was introduced by the Plaxco group.^79^ Similarly to the HPB approach for protein detection discussed in the previous section (Figure 4E), a peptidic recognition element is conjugated on both termini to two complementary PNA strands. In the absence of the target Ab, the formation of a stable PNA stem promotes the proximity of a fluorophore quencher-pair. The binding of an anti-HIV Ab, however, causes the peptide to adopt an extended conformation, thereby breaking the stem and enhancing the fluorescent signal. Such probes were termed chimeric peptide beacons (CPBs) and were later used by the Jung team to fluorescently detect influenza A subtype H1N1 viruses.^80^

**Figure 5:**
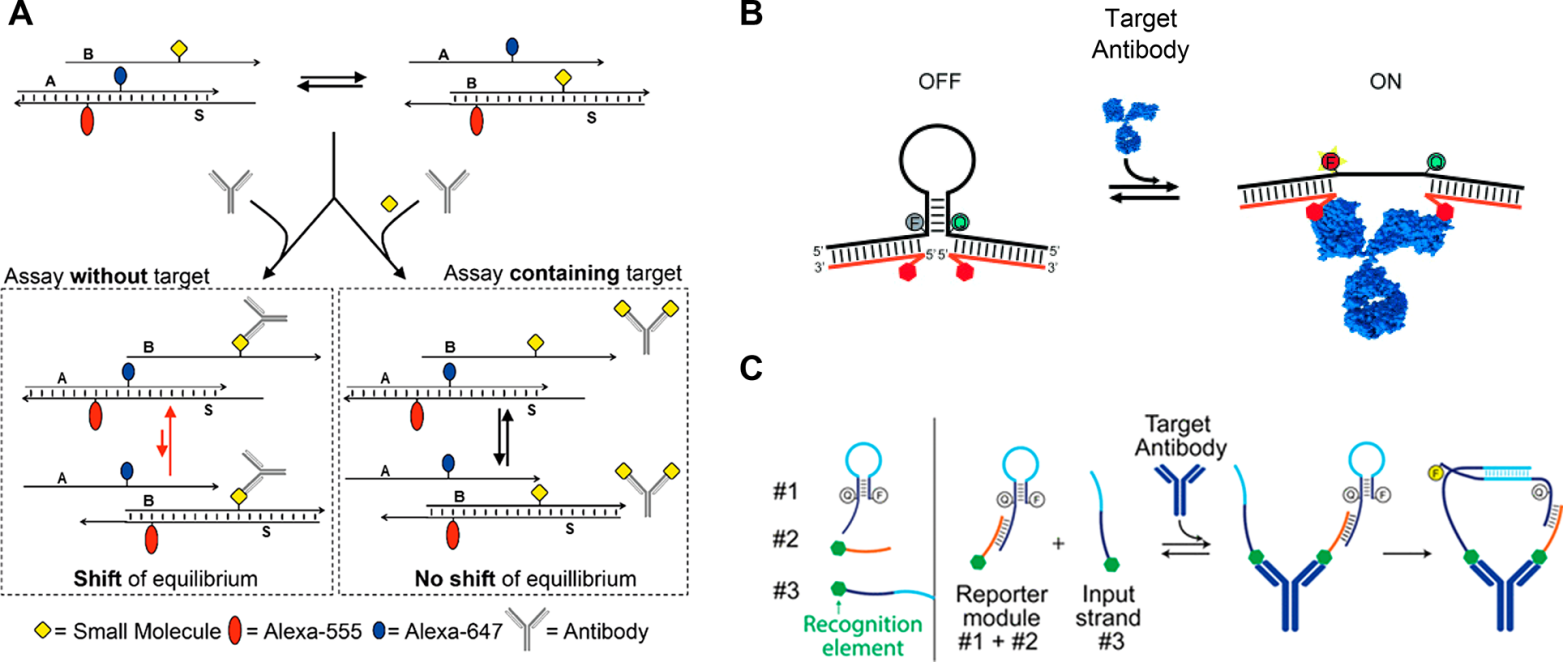
A. Operating principles of SDC assay, aimed at either Ab (left) or SM Ag (right) detection. Reproduced with permission from ref (82), published by MDPI and licensed under Creative Commons 4.0 (CC 4.0) series. B. Operating principles of Ab-switch. Adapted with permission from ref (86). Copyright 2015 John Wiley & Sons, Inc. C. A three-component design of an Ab-switch for SM Ag detection. Reproduced with permission from ref (88). Copyright 2018 American Chemical Society.

In a different approach, the Gothelf lab introduced the strand displacement competition (SDC) assay for the detection of proteins and small molecules.^81^ SDC is governed by the decrease in duplex stability following POI binding, leading to a strand displacement event accompanied by the generation of a FRET output (Figure 5A, left). This platform is composed of three strands, A, B, and S, which reach a dynamic equilibrium, highly dependent on the difference in the melting temperatures (*T*_m_) of duplexes AS and BS. Furthermore, strands A and S are appended with a FRET pair, whereas the SM Ag-of-interest is conjugated to strand B. The binding of an Ab to its specific Ag decreases the *T*_m_ of duplex BS, facilitating the displacement of the protein-bound strand and the formation of AS, accompanied by FRET (Figure 5A, left). SDC was successfully used to detect both digoxigenin–anti-digoxigenin and FA–antifolate (aFA) interactions. Furthermore, the ability to sense Abs with ODN-Ag conjugates can be further extrapolated to detect free Ab-binding SMs in solution (Figure 5A, right),^82^ aiming at implementing the method to detect SM drugs in complex biological mixtures. Following the same scheme and in the presence of free SM Ags, the Ab will be precaptured; therefore, the equilibrium will not be shifted toward AS duplex formation, resulting in low FRET. Intriguingly, a recent study from the same group challenged the mechanistic hypothesis of SDC, revealing that the increase in FRET due to displacement is a long-term effect, while a surprising kinetic effect can be detected in the initial stages of the assay.^83^ A different sensing platform exploits the advantageous hybridization chain reaction,^84^ facilitating the sensing of SM-Ags in human plasma. Detection was achieved using Cy3- and Cy5-appended hybridizing strands and monitoring the FRET signal resulting from strand elongation.^85^

In 2015, Ricci and co-workers introduced the Ab-switch strategy, relying on a conformational change induced on the sensor upon binding of the Ab target to carefully positioned Ags.^86^ Similar to molecular beacons,^87^ the fluorescence of an Ab-switch is turned on by opening a stem-loop scaffold in response to the binding of anti-digoxigenin Abs. Here, Ab binding results in a steric strain that induces stem opening and, consequently, a separation of the fluorophore–quencher pair at its termini (Figure 5B). Ab-switch was successfully expanded to detect additional selected Abs, including the clinically relevant anti-HIV p17 Ab. Revelation of the rapid and sensitive detection capabilities of this approach (<10 min and low nM, respectively) was followed by a demonstration of modularity; a sensor that operates as a molecular AND logic gate was developed, producing a fluorescent ON signal only in the presence of two different antibodies.

Initially, Ab-switch required meticulous adaptation of the distance between recognition elements and was therefore evolved into a three-component system, combining the advantages of DNA-nanoswitches with a colocalization approach.^88^ In this system, the first component is a stem-loop strand appended with a fluorophore-quencher pair as well as an overhanging strand (“tail”) (Figure 5C, #1). This tail is designed to complement a second strand appended with an Ag (#2). Strand #3 consists of an additional Ag molecule, as well as a complementary sequence to the loop in #1. Simultaneous binding of the target Ab to the two Ags on #2 and #3 induces the proximity between strands #1 and #3, thereby increasing their hybridization probability and, consequently, the opening of the stem. As in the original methodology (Figure 5B), this step is accompanied by a fluorescent output signal. A major strength of this three-component system lies in its flexibility; due to the shared geometry of IgG and IgE Abs, the system can potentially be adapted to detect any desired Ab by simply modifying the conjugated Ags. Impressively, by assigning different fluorophore–quencher pairs to each recognition element, three different Abs species were sensed simultaneously. The capabilities of DNA switches were further expanded towards the detection of SM Ags.^89^ The latter prevent the formation of the DNA switch-Ab complex and the consequent change in the emission signal.

### 3.3 Pattern-Based Detection of Different Protein Isoforms

Previously (Section 2.2.3, Figure 3), the principles underlying the function of protein surface sensors that can distinguish between different His-tagged glycoforms were discussed.^58^ By using similar design principles, the Margulies team created protein surface sensors that bind to the natural LBDs of POIs. These sensors target nonengineered proteins and could therefore be applied to identify and distinguish between isoform biomarkers in biofluids or living cells.

#### 3.3.1 Isoform Differentiation in Biofluids Using a Cross-Reactive Sensor Array

**Figure 6:**
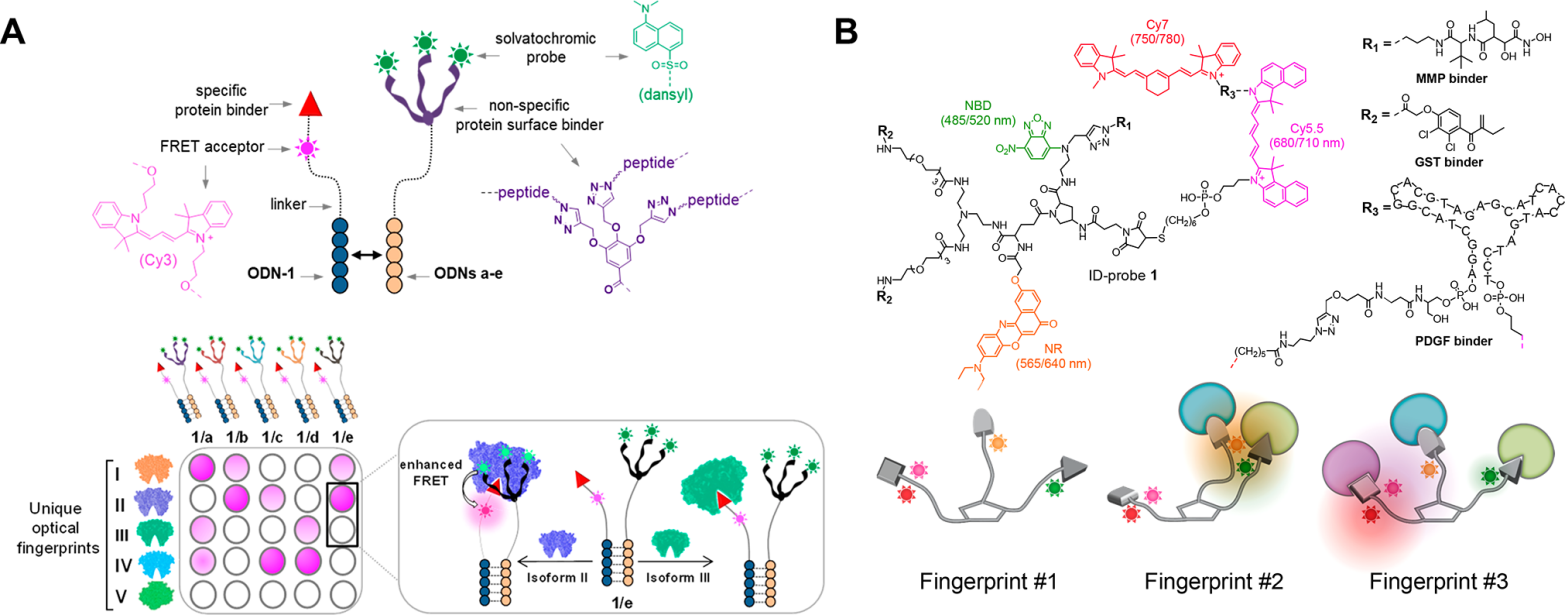
A. Analysis of an isoform population in biofluids using targeted protein receptors: structure (top), operating principles, and interactions with different isoforms (bottom). Adapted with permission from ref (77). Copyright 2014 John Wiley & Sons, Inc. B. Structure (top) and operating principles (bottom) of a pattern-generating probe that can differentiate between distinct isoform populations in living cells. Adapted with permission from ref (92). Copyright 2017 Springer Nature.

To distinguish between biomarker isoforms of glutathione S-transferase (GST) a cross-reactive sensor array (or a chemical “nose/tongue”^59,90,91^) consisting of different duplex-based protein surface sensors (Figure 6A, top), was devised.^77^ Each sensor is generated from two types of complementary strands; ODN-1 is appended with Cy3 and a bis-ethacrynic amide (bis-EA) GST inhibitor, strongly interacting with a broad spectrum of GST isoforms. The second strand is linked to a solvatochromic dye (i.e., dansyl) and a tripodal peptide, serving as the nonspecific component of the system. By modification of the peptide sequence on each strand, an array of five different sensors was generated (Figure 6A bottom, probes 1/a–e). Because different GST isoforms differ in their surface characteristics, they are expected to interact differently with each sensor and induce a distinct fluorescence response (Figure 6A bottom, isoform II vs isoform III). The differences in the emission signals result from the specific distance between the FRET donor (dansyl) and acceptor (Cy3), as well as the molecular environment of the solvatochromic dye. Using this array, GST isoforms were differentiated according to their optical fingerprints (Figure 6A bottom) even in complex biological environments such as human urine.

#### 3.3.2 Isoform Differentiation in Living Cells by a Pattern-Generating Fluorescent Molecular Probe

A more advanced isoform differentiating system, developed by the same group, consists of a unimolecular pattern-generation probe (ID-probe) able to generate unique identification (ID) fingerprints for three different isoform families: GSTs, matrix metalloproteases (MMPs), and platelet-derived growth factors (PDGFs) (Figure 6B top, ID-probe **1**).^92^ The ID-probe integrates specific binders for these families, namely, bis-EA, marimastat (MT), and an anti-PDGF aptamer, respectively. Additionally, ID-probe **1** contains four dyes: NBD, NR, Cy5, and Cy7, enabling the generation of emission patterns. In this system, the selective binding to one of the target isoforms induces nonspecific interactions between the various dyes and recognition elements and the POI’s surface. For instance, binding to GST or MMP isoforms is likely to induce an electrostatic interaction between the aptamer (the PDGF binder) and the surface of the POIs (Figure 6B bottom), affecting the fluorescence response. ID-probe **1** was applied to construct a high-throughput screening assay that can simultaneously detect (*in vitro*) inhibitors of distinct enzymes. In addition, it was utilized to discriminate between isoforms in living cells and distinguish between clinically relevant intracellular states. This showed, for the first time, the feasibility of creating molecule-size artificial “nose/tongue” devices that can operate in confined microscopic environments.

### 3.4 Creating Multivalent Protein Binders

The multivalency principle is applied in many natural receptor–ligand interactions to enhance interactions that would otherwise be weak. Whereas investigating the structural parameters affecting the binding of a monovalent ligand to its target is relatively simple, for example, by performing structure–activity relationship studies, exploring the simultaneous interactions of multiple ligands is quite challenging. Complications arise from, among other factors, various additional parameters such as the number of ligands, their orientation, and their ability to engage in cooperative binding.

Using DNA templates to study multivalency encompasses many potential benefits. The key advantage is that well-defined three-dimensional structures can be readily generated and modified to include different numbers of ligands in a site- and orientation-specific manner. Generally constructed through precise control over Watson–Crick base paring, Hoogsteen base pairing has also been used to create multivalent protein binders as elegantly demonstrated by the Hamilton team.^93,94^ Another benefit of using DNA scaffolds to probe multivalent interactions is the simplicity by which they can be labeled with a fluorescent reporter. Unlike multivalent systems based on synthetic dendrimers, liposomes, or nanoparticles,^95^ DNA self-assembly provides a simple means to fluorescently label the protein without interfering with the binding event, enabling the study of the interaction using fluorescence anisotropy. As this review spotlights applications of fluorescent ODN-SM or peptide conjugates, we will not discuss other important studies that utilized aptamers to guide the multivalent binding or outputs other than fluorescence. For a general outlook of this field, we refer the readers to a comprehensive review recently published by Seitz and co-workers.^96^

#### 3.4.1 Investigating Multivalent Protein–Ligand Interactions

**Figure 7:**
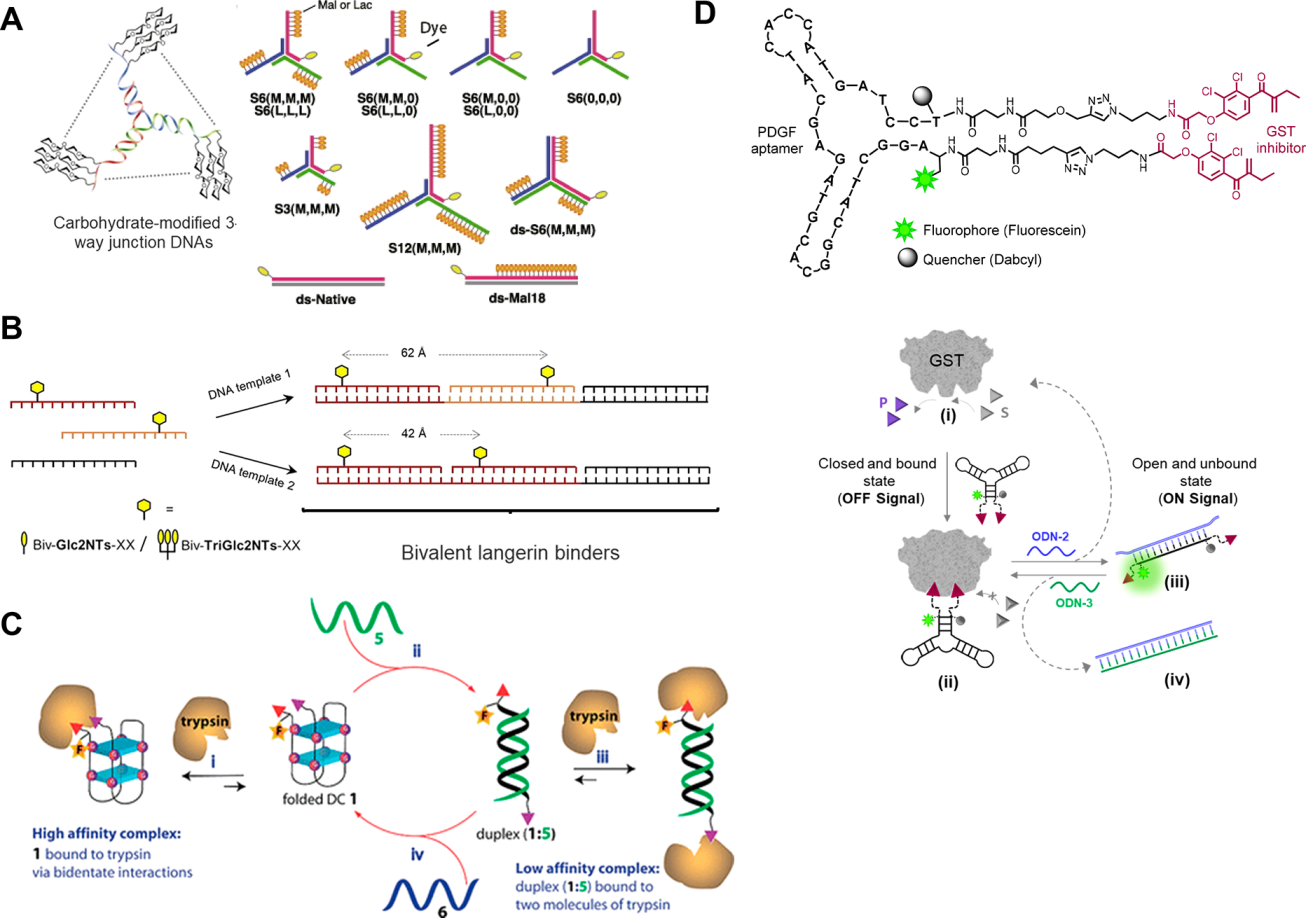
A. Left – General design of carbohydrate-modified 3-way junction DNAs. Right – Library of trigonal constructs. Adapted with permission from ref (97). Copyright 2012 Elsevier. B. Hybridization of PNA oligomers with DNA templates affords bivalent complexes that display ligands in different spacer lengths. Adapted with permission from ref (98), published by John Wiley & Sons and licensed under Creative Commons 4.0 (CC 4.0) series. C. Controlled trypsin-binding states through the addition of external DNA stimuli (green and blue strands). Reproduced with permission from ref (99). Copyright 2008 American Chemical Society. D. Molecular design (top) and operating principles (bottom) of a bivalent and reversible GST inhibitor. Adapted with permission from ref (101). Copyright 2015 American Chemical Society.

Multivalent interactions between lectins and carbohydrates were studied by the Ebara group using novel trigonal DNA–carbohydrate conjugates (Figure 7A, left).^97^ Diverging from the classical double helix, a Y-shaped architecture was explored, aiming at enhancing the affinity by controlling the spatial distribution of ligands. By including a fluorophore (Figure 7A, right), fluorescence-based assays could be used to evaluate the binding to the lectin protein model, concanavalin A (Con A). Interestingly, the spatial distribution of the carbohydrates was shown to have a higher impact on the affinity for Con A than the number of carbohydrates per arm. Furthermore, introducing rigidity to the arms by enforcing a full double helix resulted in a significant affinity decrease, while a 700-fold higher affinity was achieved with a trigonal structure containing six maltose ligands on each arm compared to a monovalent ligand.

Recently, the Seitz group introduced a rationally designed high-affinity selective binder of langerin,^98^ a lectin that mediates the internalization of pathogens. One of the objectives of this study was to offer nanomolar affinity binders that require the display of only a few glycomimetic ligands. The design of a library of DNA:PNA complexes included the presentation of either monomeric or trimeric glycan ligands in a bivalent manner, with different spacing between the two binding sites (Figure 7B). Using this straightforward approach, a Cy5-appended langerin binder facilitated the study of binding and cellular internalization in langerin-expressing cells via flow cytometry.

#### 3.4.2 Switchable Multivalent Systems

Another benefit of generating multivalent protein binders from DNA scaffolds is the ability to switch between mono- and multivalent binding modes in response to an external stimulus. With such switchable systems, fluorescence provides a convenient means to track the binding event in real-time. A prominent example of such a fluorescent switchable probe was presented by the Jayawickramarajah group (Figure 7C).^99^ A bidentate trypsin binder was generated by appending a G-quadruplex-forming strand with two SM-based binders. Switching into a monovalent binding mode was facilitated by the addition of an external stimulus (ODN 5), converting the probe into a duplex and resulting in a 20-fold affinity decrease. Furthermore, reversibility was demonstrated by the addition of ODN 6, resulting in the capture of ODN 5. Even though this specific system did not demonstrate control over trypsin activity, one can envision the potential of such structure-switching systems in stimuli-responsive drug release. In a different approach, Jayawickramarajah and co-workers combined host–guest interactions with an ODN-induced conformational switch.^100^ In this strategy, the probe is modified with a β-cyclodextrin host and a guest ligand at opposite termini, and the accessibility of the ligand is controlled by its release from encapsulation due to the addition of a complementary strand. Importantly, in both systems, fluorescent investigation of POI binding by anisotropy was facilitated by incorporating a fluorophore in the sequence.

A molecular device that combines conformational switching with control over protein activity was introduced by the Margulies team (Figure 7D, top). The device, termed a chemical transducer (CT), was designed to mediate artificial protein–protein communication between two unrelated proteins.^101^ In addition to demonstrating the ability of the CT to mediate activation of GST by another protein, the team showed that with suitable inputs, GST activity can be reversibly controlled (Figure 7D, bottom). In the closed form of the transducer, the two GST inhibitors project in the same direction, forming a high-affinity bivalent binder and inhibiting GST activity (Figure 7D, state (ii)). However, the addition of ODN-2 imposes the formation of a duplex in which the two EA groups point in opposite directions (state (iii)), transforming the CT into a weak monovalent binder and leading to the reactivation of the enzyme. The reversibility of this system was demonstrated by adding ODN-3, which captures ODN-2 (state (iv)), leading to GST reinhibition. By appending the termini of the CT with a fluorophore (FAM) and a quencher (dabcyl), the two conformations of the transducer could be distinguished by detecting an ON or OFF signal.

#### 3.4.3 Multivalent Recognition of Cancer Cells

**Figure 8:**
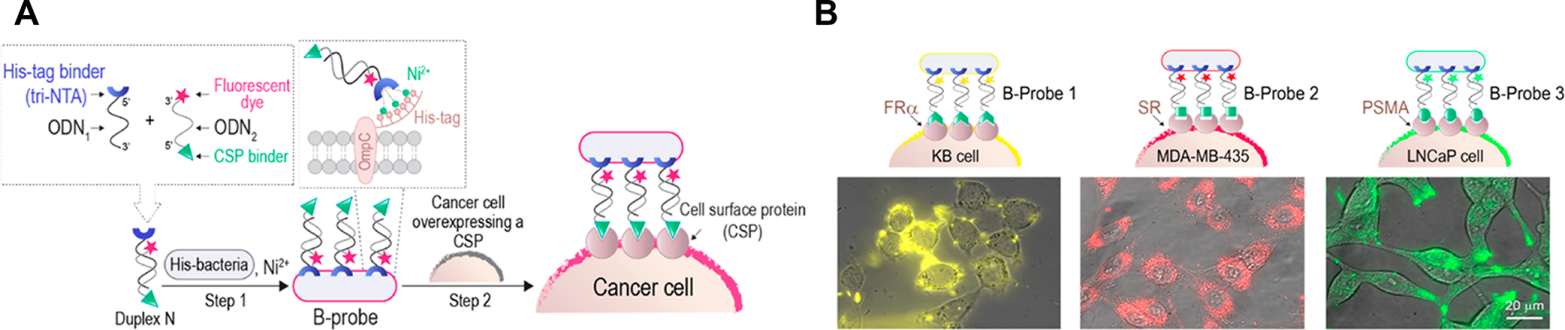
A. Generation of B-probes and their multivalent fluorescent labeling of various cancer cells. B. Fluorescent images of cancer cells after treatment with the B-probes, appended with specific CSP ligands and different fluorescent dyes: TAMRA (yellow), Cy5 (red), and FAM (green). Adapted with permission from ref (102), published by Elsevier and licensed under Creative Commons 4.0 (CC 4.0) series.

The Margulies team utilized their method for decorating bacteria with modified DNA strands in a programmable and reversible manner (Figure 2C, Section 2.2) to obtain “living bacterial probes” (B-probes) that can fluorescently label different types of cancer cells (Figure 8).^54,102^ Such B-probes were generated by attaching modified duplexes to His-tagged OmpC proteins of *E. coli*. The duplexes include a His-tag binding strand (tri-NTA-ODN-1, Figure 8A) and a complementary strand (ODN-2) bearing a dye of choice and a CSP ligand.^54^ Selective labeling of specific cancer cells that overexpress distinct CSPs (FR, SR, or PSMA) was achieved using three different B-probes (Figure 8B).^102^ The high efficiency by which the B-probes labeled the cancer cells is attributed to two main factors: The first is the numerous ligands covering the bacterial scaffolds, enabling the B-probes to engage in multivalent interactions with the cancer cells. The second factor is the large number of fluorophores decorating each bacterium, enabling individual B-probes to generate a strong emission signal.

## 4 Conclusions and Prospects

This review highlights the various advantages of fluorescently investigating proteins through the use of ODN-synthetic ligand conjugates. One highly favorable attribute of such systems lies in their flexible nature: careful design of complementary strands facilitates the incorporation of reversibility, sensitivity, and enhanced affinity into the molecular device. This not only simplifies the synthetic challenges associated with integrating a wide range of fluorescent dyes and protein binders on a single molecular platform—but also provides a simple means to control their number and orientation.

Focusing in this review on SM or peptide ligands responsible for directing DNA-based probes to the POI, a diverse array of platforms was described. Fluorescent labeling of POIs, for example, can be driven by the ability of single strands to hybridize in a predetermined manner. Moreover, the scope of the ODN-guided fluorescent labeling is not restricted to purified proteins but can also be elegantly applied to labeling membrane proteins in living cells. Furthermore, navigation of the fluorescent probe to its POI can be achieved by targeting its LBD or genetically modifying the protein with a small peptide tag. Fluorescence-based sensing of various POIs was also explored using responsive probes in which the fluorescent output changes upon ligand–protein binding. Various therapeutically relevant proteins, ranging from low molecular weight to whole Abs, were shown to be efficiently detected using ODN-synthetic ligand conjugates as well as subtle protein surface variations such as glycosylation. In addition, tight control over the number and position of synthetic ligands was shown to facilitate the fluorescent study of multivalency, a feature that can be elegantly incorporated to generate high-affinity and ultrabright diagnostic tools that can be straightforwardly followed by fluorescence polarization.

We hope this review will showcase some notable applications of ODN-ligand conjugates in fluorescent labeling and sensing as well as the vast potential of using such systems for diverse biological studies and chemical investigations. Although this summary presents many versatile examples, there are still challenges that need to be addressed: First, the conjugation of ODNs to synthetic ligands often reduces the affinity of the ligands toward the POI. Although incorporating multivalency into the design can enhance binding, conducting a systematic study on the optimal spacer length would be highly beneficial. Second, utilizing the use of ODN-based probes inside living cells may prove to be challenging due to their low permeability, as well as degradation by cellular nucleases. These two issues should be addressed by developing efficient delivery systems as well as by chemically modifying the DNA backbone to resist intracellular digestion. Improvements in these features, together with the generation of low-background fluorescent dyes compatible with living cells, could pave the way toward novel, highly efficient diagnostic and therapeutic platforms.

## Abbreviations

ODN: Oligodeoxynucleotide
SM: Small-molecule
POI: Protein of interest
LBD: Ligand binding domain
DTS: DNA-templated synthesis
DPAL: DNA-programmed photoaffinity labeling
BP: Binding probe
CP: Capture probe
FP: Fluorescent strand
His-tag: Hexahistidine tag
NTA: Nitrilotriacetic acid
DTPC: DNA-templated protein conjugation
IgG: Immunoglobulin G
NHS: *N*-Hydroxysuccinimide
CSPs: Cell surface proteins
EGFR: Epidermal growth factor receptor
PNA: Peptide nucleic acid
OmpC: Outer membrane protein C
STORM: Stochastic optical reconstruction microscopy
An-BA: Anthracene-boronic acid
FRET: Förster resonance energy transfer
Ab: Antibody
Ag: Antigen
Exos: Exonucleases
FR: Folate receptor
QR: Quinaldine red
FA: Folic acid/folate
SA: Streptavidin
MB: Molecular beacon
crRNA: CRISPR RNAHPB
HPB: Hairpin peptide beacon
CPB: Chimeric peptide beacon
SDC: Strand displacement competition
*T*_m_: Melting temperatures
GST: Glutathione S-transferase
bis-EA: Bis-ethacrynic amide
MMPs: Matrix metalloproteases
PDGFs: Platelet-derived growth factors
Con A: Concanavalin A
CT: Chemical transducer
B-probes: (Living) bacterial probes
SR: Sigma receptor
PSMA: Prostate-specific membrane antigen
